# The British Journals, &c.: Anatomy and Physiology

**Published:** 1842-10

**Authors:** 


### III. THE BRITISH JOURNALS.

### ANATOMY AND PHYSIOLOGY.

On the Physiology of Secretion and Absorption. By Messrs. Goodsir and Bowman. [We have great pleasure in directing the attention of our readers to the recent observations of Mr. Goodsir and Mr. Bowman (of which we subjoin the general results), as constituting a most important accession to our knowledge of the two functions by which the living organism is brought into relation with the world around. These observations are also of peculiar value, as demonstrating the utility of the microscope in anatomico-physiological research, a fact which the differences of opinion amongst observers, in regard to many questions of great delicacy, have led many to doubt.

We mentioned in our last Number (p. 291-3) the very probable theory (which, though extended and made more definite by Henle, was first propounded by Purkinje,) that the nucleated epithelium-cells of the gland-ducts are the real agents in the secreting process, elaborating from the blood certain of its constituents, just as do other cells, but subsequently discharging these by rupture or other modes; and we pointed out that, on this view, it is not possible to draw a strict line between secretion and nutrition. The first of Mr. Goodsir's papers may be considered as adducing almost demonstrative evidence of this theory (to which Mr. Bowman's observations on the fatty degeneration of the liver afforded no small support); whilst the second extends it to the process of selective absorption also. Mr. Bowman's researches into the minute anatomy of the kidney have most successfully determined a long-disputed question as to the nature and connexions of the Malpighian bodies; whilst they have also thrown great light on the mechanism (so to speak) of the renal secretion.

It is necessary that we should draw the attention of our readers *in limine* to an important distinction between *selective absorption* and *imbibition*, and to a difference of a similar kind between true *secretion* and *fluid exhalation*. Imbibition and exhalation of fluid are changes of a merely physical nature, depending upon the amount of fluid in the vessels and tissues of the body, and upon the permeability of these; and they take place, as is well known, in the dead as in the living body. Not only does water thus transude, but various substances which are held in complete solution in it, especially those of a saline character; and the quantity and nature of the fluids of the body may thus undergo a considerable change, without any purely vital function having any direct connexion with it. This change we believe to be chiefly accomplished through the medium of the blood-vessels, which absorb, through the capillary plexus of the villi of the intestines, perhaps a larger amount of fluid than is taken in by the lacteals; and which, as we shall presently see, get rid of what is superfluous, by an exhalation through the thin-walled capillary plexus of the Malpighian bodies of the kidney. On the other hand, *selective absorption* and *selective secretion* both seem to be performed by the agency of cells, which, during the progress of their development and growth, appropriate certain substances with which they may be in relation; and, when their term of existence is over (which is longer or shorter in different cases), they discharge their contents, in the one case into the neighbourhood of the absorbent vessels, and in the other into the channels of excretion. Of the reason why certain cells should perform these functions, we know no more than we do why certain others should be transformed into the several tissues of the body; but the one process is just as comprehensible as the other.]

1. *On the Ultimate Secreting Structure, and on the Laws of its Function.* By John Goodsir, Conservator of the Museum of the Royal College of Surgeons, Edinburgh.

After a brief statement of the history of opinion on this subject, the author remarks: "No one, so far as I am aware, has proved that secretion takes place within the nucleated cell, or has pointed out the intimate nature of the changes which go on in a secreting organ, during the performance of its function. It is the object of the present communication to supply this deficiency in physiological science." He then enumerates several instances in which he has found the secreting surface covered by cells that evidently contained the characteristic elements of the peculiar secretion. Amongst others we may mention the lining membrane of the ink-bag of the cuttle-fish—the terminal sacculi of the hepatic organs of numerous radiata, mollusca, articulata, and vertebrata—the generative cæca, whose cells contain spermatozoa—the colour-secreting glandulæ in the margin of the mantle of aplysia and janthina—and the mammary gland of the bitch. In all these instances, the character of the secreted product was easily recognizable by its appearance. In others, the secreted products are too transparent and colourless to be distinguished by the microscope; and we do not possess chemical tests of sufficient delicacy for the detection of such minute quantities. But the identity of the changes that can be proved to occur in the cellular elements of the organs that form the colourless secretions, together with the general argument drawn from the analogy, leave no doubt that the plan is the same.

The author then goes on to state the laws which he has deduced from numerous observations, as regulating the original formation, the development, and the disappearance of the primary organ. He contents himself in the present paper with giving two illustrations of the mode in which these laws apply to particular instances. One of these illustrations is drawn from the testicle of *squalus cornubicus*, which is taken by the author as the type of a class of glands in which the secretion is formed in the cells of their parenchyma; amongst which we presume that the human liver is to be included. The following is his summary of the process:

"1. The glandular parenchyma is in a constant state of change, passing through stages of development, maturity, and atrophy.

"2. This state of change is contemporaneous with and proportional to the formation of the secretion; being rapid when the latter is profuse, and *vice versâ*.

"3. There are not, as has hitherto been supposed, two vital processes going on at the same time in the gland, growth and secretion; but only one, viz. growth. The only difference between this kind of growth and that which occurs in other organs being, that a portion of the product is, from the anatomical condition of the part, thrown out of the system.

"4. The vital formative process which goes on in a gland is regulated by the anatomical laws of other primitive cellular parts.

"5. An acinus is at first a single nucleated cell. From the nucleus of this cell others are produced. From these, again, others arise in the same manner. The parent cell, however, does not dissolve away, but remains as a covering to the whole mass, and is appended to the extremity of the duct. Its cavity, therefore, as a consequence of its mode of development, has no communication with the duct.—The original parent-cell now begins to dissolve away, or to burst into the duct at a period when its contents have attained their full maturity. This period varies in different glands, according to a law or laws impressed upon each of them.

"6. In the gland there are a number of points from which acini are developed, as from so many centres. These I denominate the germinal spots of the gland.

"7. The secretion of a gland is not the product of the parent-cell of the acinus, but of its included mass of cells. The parent-cell or vesicle may be denominated the primary cell; its included nucleated cells, after they have become primary secreting cells, may be denominated secondary cells of the acinus.

"8. The matter which passes off by a duct of a gland may be, 1st, a true secretion, that is, matter formed in the primary secreting cell cavities; or 2d, a mixture of a fluid formed in these cell cavities, with the developed or undeveloped nuclei of the cells themselves; and 3d, it may be a number of secondary cells passing out entire."

The author then gives a similar history of the process of secretion as it takes place in the cells lining the hepatic cæca of *carcinus mœnas*, which may be taken as the type of the follicular glands; such cells may be regarded in the light of an epithelium, as in the tubes of the human kidney.

"1. Each follicle is virtually permanent, but actually in a constant state of development and growth.

"2. This growth is contemporaneous with the function of the gland, that function being merely a part of the growth, and a consequence of the circumstances under which it occurs.

"3. Each follicle possesses a germinal spot situated at its blind extremity.

"4. The vital action of some follicles is continuous, the germinal spot in each never ceasing to develope nucleated cells, which take on the action of and become primary secreting cells as they advance along the follicle. The action of other follicles is periodical.

"5. I have not been able to satisfy myself, but I am inclined to believe, that the wall of the follicle also is in a state of progressive growth, acquiring additions to its length at its blind extremity, and becoming absorbed at its attached extremity. A progressive growth of this kind would account for the steady advance of its attached contents, and would also place the wall of the follicle in the same category with the primary vesicle or wall of the acinus in the vesicular glands.

"6. The primary secreting cells of the follicle are not always isolated. They are sometimes arranged in groups; and when they are so, each group is inclosed within its parent cell, the group of cells advancing in development according to its position in the follicle, but never exceeding a particular size in each follicle."

The author, lastly, points out the analogy between this history of the growth of a gland during its state of functional activity, and that of its embryonic development; and he closes with two important suggestions: 1st, that the follicle, like the acinus, is originally a single nucleated cell, and is the source of the germinal spot which plays so important a part in the subsequent operations; 2d, that the ducts of glands are intercellular passages, like those which contain the products of secretion in plants.—*Transactions of the Royal Society of Edinburgh*, 1842.

II. *On the Structure of the Intestinal Villi in Man and certain of the Mammalia, with some observations on Digestion, and the Absorption of Chyle.* By JOHN GOODSIR.

Having fed a dog with oatmeal, milk, and butter, the author examined the intestinal villi three hours afterwards, when the lacteals were turgid with chyle, and the gut full of milky chyme mingled with a bilious-looking fluid. In the white portion of the fluid, which was situated principally towards the mucous membrane, numerous epithelium cells were found; some of which had evidently (from their form) been detached from the surface of the villi, whilst others had been thrown off from the interior of the follicles of Lieberkuhn. The villi were turgid, and destitute of epithelium except around their bases. Each villus was covered by a very fine smooth membrane, continuous with what Mr. Bowman terms the basement membrane of the mucous surface, which is reflected into the follicles. The villi were semi-transparent, except at their free or bulbous extremities, where they were white and nearly opaque. The summit of each villus was crowded, beneath the enveloping membrane, with a number of perfectly-spherical vesicles, varying in size from 1-1000th to 1-2000th of an inch; the matter in the interior of which had an opalescent milky appearance. At the part where these vesicles approached the granular texture of the substance of the villus, minute granular or oily particles were situated in great numbers. The trunks of two lacteals could be easily traced up the centre of the villus; and as they approached the vesicular mass, they subdivided and looped; but in no instance could they be seen to communicate directly with any of the vesicles.

These vesicles can scarcely be considered in any other light than as cells, whose lives have but a very brief duration, selecting from and appropriating the materials in contact with the surface of the villi into their own substance, and then liberating these, by solution or disruption of the cell-wall, in a situation where they can be absorbed by the lacteals. When the gut contains no more chyme, the development of new vesicles ceases, the lacteals empty themselves, and the villi become flaccid. During this interval of repose, the epithelium is renewed, for the protection of the surface of the villi, and for the secreting function of the follicles of Lieberkuhn. It is considered by Mr. Goodsir that the epithelium-cells have their origin in certain nuclei which he detects scattered through the basement membrane.

There appears to be a strong resemblance between the process of absorption in animals, as thus explained, and that which takes place in plants through the medium of the spongiole.—*Edin. New Philosophical Journal*, July, 1842.

III. *On the Structure and Use of the Malpighian Bodies of the Kidney, with observations on the Circulation through that Gland.* By William Bowman, F.R.S. Demonstrator of Anatomy in King's College, London.

[The Malpighian bodies have excited much interest amongst anatomists, from the time of their first discovery; but no satisfactory account has yet been given of their structure and vascular connexions. Still less has their true relation with the uriniferous tubes been ascertained; in fact, Müller and Huschke, two of the latest inquirers on the subject, have positively denied the existence of any such relation. The following is the account of Mr. Bowman's researches given in the Proceedings of the Royal Society.]

"The author describes the results of his examination of the structure and connexions of the Malpighian bodies of the kidney in different tribes of vertebrata, and shows that they consist essentially of a small mass of vessels contained within dilated extremities of the convoluted uriniferous tubes. The tubes themselves consist of an outer transparent membrane (termed by the author the *basement membrane*) lined by epithelium. This basement membrane, where it is expanded over the tuft of vessels, constitutes the capsule described by Müller. The epithelium lining the uriniferous tube is altered in its character where the tube is continuous with the capsule, being there more transparent, and furnished with cilia, which, in the frog, may be seen, for many hours after death, in very active motion, directing a current down the tube. Farther within the capsule the epithelium is excessively delicate, and even in many cases absent. The renal artery, with the exception of a few branches given off to the capsule, surrounding fat and coats of the larger blood-vessels, divides itself into minute twigs, which are, the afferent vessels of the Malpighian tufts. After it has pierced the capsule, the twig dilates, and suddenly divides and subdivides itself into several minute branches, terminating in convoluted capillaries, which are collected in the form of a ball; and from the interior of the ball the solitary efferent vessel emerges, passing out of the capsule by the side of the single afferent vessel. This ball lies loose and bare in the capsule, being attached to it only by its afferent and efferent vessel, and is divided into as many lobes as there are primary subdivisions of the afferent vessel; and every vessel composing it is bare and uncovered, an arrangement of which the economy presents no other example. The efferent vessels, on leaving the Malpighian bodies, enter separately the plexus of capillaries surrounding the uriniferous tubes, and supply that plexus with blood. The blood of the vasa vasorum also probably enters this plexus. The plexus itself lies on the outside of the tubes, on the deep surface of the membrane which furnishes the secretion; and from it the renal vein arises by numerous radicles.

Thus the blood, in its course through the kidney, passes through two distinct systems of capillary vessels; first, through that within the extremities of the uriniferous tubes, and secondly, through that on the exterior of these tubes. The author points out striking differences between these two systems. He also describes collectively, under the name of *Portal System of the Kidney*, all the solitary efferent vessels of the Malpighian bodies, and compares them with the portal system of the liver, both serving to convey blood between two capillary systems. In the latter a trunk is formed merely for the convenience of transport, the two systems it connects being far apart. But a portion even of this has no venous trunk, viz. that furnished by the capillaries of the hepatic artery throughout the liver, which pour themselves either into the terminal branches of the portal vein, or else directly into the portal-hepatic capillary plexus. On the other hand, in the kidney, the efferent vessels of the Malpighian bodies, situated near the medullary cones, having to supply the plexus of the cones, which is at some little distance, are often large, and divide themselves after the manner of an artery. They are portal veins in miniature. In further confirmation of his view of the existence of a true portal system in the kidney of the higher order of animals, where it has never hitherto been suspected, the author describes his observations on the circulation through the kidney of the boa constrictor, an animal which affords a good example of those in which portal blood derived from the hinder part of the body traverses the kidney. He shows that here the Malpighian bodies are supplied, as elsewhere, by the artery, and that their efferent vessels are radicles of the vena portæ within the organ, and join its branches as they are dividing to form the plexus surrounding the tubes; thus corresponding with the hepatic origin of the great vena portæ. In other words, the vena portæ is an appendage to the efferent vessels of the Malpighian bodies, and aids them in supplying blood to the plexus of the tubes. Thus in this variety of the kidney, as in the liver, there is an internal as well as an external origin of the portal system; while in the kidney of the higher animals, this system has only an internal or renal origin, viz., that from the Malpighian bodies.

A detail of the results of injection by the arteries, veins, and ducts, is then given, and they are shown to accord with the preceding description. Many varieties in the Malpighian bodies in different animals are also pointed out, especially as regards their size.

The author then proceeds to found on his previous observations, and on other grounds, a theory of a double function of the kidney. He conceives that the aqueous portion of the secretion is furnished by the Malpighian bodies, and its characteristic proximate principles by the walls of the tubes. After giving in detail his reasons for entertaining this view, he concludes by referring to the striking analogy between the liver and kidney both in structure and function, and by expressing his belief, first, that diuretic medicines act specially on the Malpighian bodies, and that many substances, especially salts, which when taken into the system have a tendency to pass off by the kidneys with rapidity, in reality escape through the Malpighian bodies; secondly, that certain morbid products occasionally found in the urine, such as sugar, albumen, and the red particles of the blood, also, in all probability, pass off through this bare system of capillaries."—*Proceedings of the Royal Society*, No. lii. *Feb.* 3, 1842.

Guided by the discovery of the normal anatomy and physiology of this organ, Mr. Bowman is now engaged in the elucidation of its morbid structure in various diseases, especially the morbus Brightii; and we can scarcely doubt that he will be equally successful. Without committing himself to any premature opinions, he informs us that the scattered red spots which are frequently seen in the cortical substance during the earlier stages of the disease (when blood is often passed with the urine, and many circumstances contribute to prove that the kidneys are in a state of sanguineous turgescence,) are certainly *not enlarged Malpighian bodies*, as they have been recently stated to be, but convoluted uriniferous tubes filled with blood that had burst into them from the gorged Malpighian tufts at their extremities. They are seen on the surface where no Malpighian bodies exist; and they are much larger than these could be supposed to become under any amount of distention.

[In conclusion, we again desire to call the attention of our readers to the papers themselves, and especially to the last. It is one of the most complete and satisfactory elucidations of an obscure question, with which we are acquainted, and is a model for all similar inquiries.]

---

Best Mode of conducting Physiological Researches. The author is opposed to that school of physiologists called *experimental*, whose rule is to withhold belief from every deduction drawn from anatomical structure, and to consider such deduction, hypothetical and unproved, until they have been over and over again tested on living animals, He, on the contrary, holds that, by drawing inferences from the anatomical structure, and adopting as our guides certain acknowledged principles acquired from the same source, we might obtain conclusions the truth of which the most sceptical could scarcely deny. The author, with considerable ingenuity, illustrates his meaning as follows:

1. Reason alone suggests that a nerve of motion should be anatomically distinct from a nerve of sensation. As these influences travel in contrary directions—that which induces muscular action being propagated *outwardly* from the brain, and that which gives rise to sensation being conveyed *inwardly* to the brain—it surely appears highly probable, at least, that each property must belong to a distinct structure.

2. The ninth pair has long been considered a motor nerve. Let us enquire what has led former physiologists to form that opinion. This nerve supplies the tongue, an organ endowed with motion and with sensibility: but it is remarkable that it is limited in its distribution to the muscular substance alone, and that it avoids sending any of its branches to the surface, which is therefore supplied by a different nerve. Now this circumstance, viz. that the ninth pair is limited to the supplying of *muscles*, carries to my mind as distinct a proof that it is subservient to motion, and that it has been correctly called the motor of the tongue, as the origin and insertion of the biceps muscle of the arm do, that the action of its fibres is to bend the forearm of the arm.

3. If we observe the peculiar structure, and also the exact point of origin, of the ninth pair, we shall be convinced that it is in truth an anterior root of a spinal nerve. In no one character can we recognise a difference between them. In short, the ninth nerve is in every point of view like a spinal nerve, which is deficient in a posterior root. May we not therefore infer that the anterior roots of the spinal nerves have a similar function to that of the ninth pair: in other words that they are nerves of motion?

4. Guided by these views, which naturally lead us to the belief that the whole line of nerves from the ninth downwards, arising from the anterior column of the spinal marrow, are subservient to motion, we look with natural curiosity to those nerves which originate from the same column prolonged into the brain.

The first nerve which meets us is the sixth pair, a nerve that goes to a muscle alone; the next is the third pair, which likewise goes to muscles exclusively. Now in the same manner as it was inferred from the distribution of the ninth pair, that its function was to confer motion, so it may be concluded that these two nerves are destined for the same purpose. Indeed the fact cannot be forgotten, that to one of them the name *motor oculi* has been applied for centuries past. The course of observation hitherto pursued consequently leads us to infer, that the whole series of nerves arising from the anterior column of the spinal marrow, from the third of the brain to the lowest nerve of the cauda equina, is destined for the giving of motor pewer.

So much for the uses of the anterior roots; let us next briefly attend to those of the posterior roots.

5. The fifth cerebral or trigeminus nerve is chiefly remarkable, in its anatomical character, for having an exact resemblance to the spinal nerves. This resemblance, it may be remarked, attracted the attention of anatomists for some time before the recent discoveries in the nervous system were thought of. The peculiar character which distinguishes the fifth from all the other nerves of the brain, is that it originates by two roots,—one of which corresponds with the anterior roots of the spinal nerves in having no ganglion upon it, while the other roots has a ganglion exactly similar in structure to that formed on the posterior roots of the spinal nerves. Our line of argument therefore, leads us to inquire whether, in the anatomical arrangement of the roots of the fifth pair, anything presents itself which can throw light on their distinct endowments. Now there is a circumstance in the anatomy of this nerve which is greatly in favour of our obtaining the desired knowledge. Instead of both its roots being of equal dimensions, that which has a ganglion, is about four times as large as that which is without one; and the former can therefore be traced to many parts of the head where it is not accompanied by the latter. If we consider the lesser roots, the analogue of the anterior roots of the spinal nerves, in the first place, and trace it to its destination, we find that it is distributed exclusively to muscles—it is the nerve proper to the muscles of the jaws. Indeed Paletta, resting on the circumstance of this root not going exclusively to the muscles, so far anticipated the present views in regard to the physiology of the fifth, as to call this root a motor nerve, and to represent it as the nerve on which *trismus* depended. Accordingly, the observation of the simple distribution of this smaller root of the fifth sanctions the conclusion that, inasmuch as it resembles the anterior roots of the spinal nerves, and I may add, the ninth pair, as well as the sixth, and the third, in its structure, it is in all probability identical with them in function—namely, in being a nerve of motion.

I proceed next to the larger root, which from having a ganglion resembles the posterior roots of the spinal nerves; and will endeavour to show how far the distribution of its branches tends to elucidate the nature of its functions.

What principally strikes an anatomist, when surveying the course of the several great branches derived from this larger root, is that they supply numerous parts of the head where no muscles exist, and when there are only sensible surfaces. I will put aside as doubtful the branches which emerge upon the face, since the muscles of the features appear to be supplied by these branches in common with the portio dura.

But taking other branches, we find that several pass to localities where no muscles are situated, and where of course we cannot suppose that nerves of motion should be sent. For example, we have the cavities of the nose liberally supplied with branches proceeding from the ganglionic root; we have branches of considerable size from the same origin going to the hard palate: branches of the same kind go to the teeth: and lastly, there is a large branch, the proper destination of which is manifestly the sensible surface of the tongue.

These facts, coupled with the circumstances that the whole extent of the integuments of the head, (except a part behind, which is supplied by spinal nerves), has branches of this root sent to them, afford by themselves strong presumptive evidence that the office of the ganglionic root of the fifth pair is to confer sensation as distinct from motion.

Now, if the inferences stated above be thought justly deducible from the premises, they may be applied legitimately to the explanation of the functions possessed by the posterior roots of the spinal nerves. We are thus led to conclude that, as the function of the larger or ganglionic root of the fifth pair is to bestow sensation, so it is the function of the posterior or ganglionic roots of the spinal nerves also to bestow sensation.

In this simple manner have we been brought, without the performance of a single experiment on a brute creature, but by relying exclusively on anatomy as our guide, to educe the beautiful truth, that the anterior roots of the spinal nerves, including their analogies in the encephalon, are subservient to motion, and the posterior roots of the spinal nerves, together with the ganglionic root of the trigeminus, to sensation.—Mr. Shaw, *Medico-Chirurgical Review*, No. 72, *April*, 1842.

---

The smallest Blood-corpuscles of Mammals. In a paper read at the Zoological Society of London, Aug. 9, 1842, Mr. Gulliver remarked that, before his discovery of the singularly minute size of the blood-discs of the musk-deer, (See Med. Chir. Trans. v. 23—Dublin Medical Press, Nov. 27, 1839—Philos. Magaz. Dec. 1, 1839,) those of the goat were the smallest known; and he announced that the blood-corpuscles of the ibex are slightly smaller than those of the goat, and therefore intermediate in size to the corpuscles of the goat, and those of the musk-deer. The following average measurements of the discs were given in fractions of an English inch.

Goat (*Capra Hircus, Linn.*) .................................. $\frac{1}{6366}$

Ibex (*Capra Caucasica ?*) .................................. $\frac{1}{6858}$

Musk-deer (*Moschus Javanicus, Pallas,* .......................... $\frac{1}{12325}$ Geo. Gulliver, F.R.S.

ON THE FIGURE OF THE OVAL BLOOD-CORPUSCLES OF VERTEBRATA. Though the usual figure of the blood-discs of the camelidæ, and of the oviparous vertebrata is an ellipse, of which the long diameter is from one and a half to twice the short diameter, Mr. Gulliver shows that there are many exceptions to this rule. In other words, the corpuscles may either be comparatively long and narrow, or short and broad. Of the former shape, M. Mandl sometime ago stated, that the corpuscles of the crocodilidæ were a singular example, since he found that the length of the corpuscles of *crocodilus lucius* was nearly three times as much as their breadth. Subsequently, (Proc. Zool. Soc. Nov. 10, 1840,) however, Mr. Gulliver found that the blood-discs of *crocodilus acutus*, and of *champa fissipes* had the more common elliptical figure first mentioned.

As examples of the elongated ellipse he has since pointed out the corpuscles of the snowy owl, passenger-pigeon, great butcher-bird, nightingale, and snow-bunting; and of the short and broad ellipse, the corpuscles of the Java sparrow and of some other granivorous birds.

Lastly, the corpuscles of the British ophidian reptiles are examples both of the ordinary and of the more elongated ellipse; for while the blood-discs of the slow-worm are long and narrow in figure, those of the snake and viper do not differ from the common shape, as the following average measurements in fractions of an English inch will show.

| | Long diameter | Short diameter |
|---|---|---|
| Slow-worm (*Anguis fragilis*, Linn.) | $\frac{1}{1178}$ | $\frac{1}{2666}$ |
| Snake (*Natrix torquata*, Ray) | $\frac{1}{1371}$ | $\frac{1}{2157}$ |
| Viper (*Coluber Berus*, Linn.) | $\frac{1}{1274}$ | $\frac{1}{1800}$ |

From a paper read by Mr. GULLIVER at the Meeting of the *Zoological Society of London*, August 9, 1842.

---

ON THE LYMPH-GLOBULES OF BIRDS. In birds, as in mammals, the lymph-globules are rather smaller than the pale globules of the blood; yet, since the time of Hewson, who describes the lymph particles of birds as oval, like the nuclei of the blood-discs, the description of the lymph-globules of birds has been drawn from the pale globules of the blood.

Mr. Gulliver figures the particles obtained from the lymphatic glands of birds as globular, which corresponds with his observations on the lymph-globules of the camelidæ and of the musk-deer. (Lancet, 1840-41, and Med. Chir. Trans. v. 23.) For numerous measurements of the lymph-globules of birds the original paper may be referred to. The pus-globules of the camelidæ are also circular.—GEORGE GULLIVER, F.R.S.; *Lond. and Edinb. Phil. Mag., June*, 1842.

---

MUSCULAR FIBRE OF THE GULLET. In a paper read at the Zoological Society, June 14, 1842, Mr. Gulliver mentioned the following as some of the results of his inquiries.

1. In man, the quadrumana, viverridæ, felidæ, the porpoise, the horse, &c., the muscular fibre of animal life terminates on the gullet at a greater or less distance before it reaches the stomach.

2. In the insectivorous feræ, in the ursidæ, mustelidæ, ruminantia, rodentia, &c., the muscular fibre of animal life runs along or forms a complete investment to the entire length of the gullet, and sometimes extends a short distance on the cardiac end of the stomach.

3. In birds and reptiles the gullet has no covering of the muscular fibre of animal life.

4. The statement therefore of Professor Müller, that "the third act of deglutition is perfectly involuntary, being performed by the muscular fibres of the œsophagus, which are not in the slightest degree capable of voluntary motion," is regarded by Mr. Gulliver as applicable to some animals, but not to others, especially those referred to in part 2.

On the Nuclei of the Blood-corpuscles of Vertebrata. The author gives two figures to illustrate the effects of several reagents, and especially of repeated washing with water, till all the colouring matter is removed, on the corpuscles of mammalia and of the lower vertebrata. The corpuscles of man for example, as Mr. Gulliver had formerly stated, are reduced about one third or one fourth in size after completely removing all their colouring matter by repeated additions of large quantities of water. These washed corpuscles appear very faint and pellucid, presenting no appearance of a nucleus, even when treated with acids and other reagents; nor do the washed blood-discs agree in any respect with the particles which have commonly been described as the nuclei of the blood-corpuscles. Now when all the colouring matter is washed away in like manner from the corpuscles of any of the lower vertebrata, both the envelopes and nuclei remain, and are plainly distinct parts, both appearing circular; although the nucleus, when exposed by acetic acid, has an oval figure. The effect of acids is equally different on the blood-discs of mammals, and on the discs of the lower vertebrate animals; and the oval corpuscles of the camelidæ agree in structure with the corpuscles of other mammals.— G. Gulliver, f.r.s.; *Lond. and Edin. Phil. Mag. for Aug.* 1842.

---

Structure of Fibrin. The red clots of fibrin found in the dead body inclose corpuscles similar, though of a ruddy colour, to the pale organic germs depicted by Mr. Gulliver in colourless clots of fibrin. (Appendix to Gerber's Anatomy.) Both kinds of corpuscles may be altered blood-discs; but if so, it is remarkable that both the ruddy and pale organic germs of fibrinous clots must have undergone great changes in form and chemical characters; these germs exhibit nuclei when treated with acetic acid, &c., while precisely the same treatment does not show any nuclei in the free or floating blood-discs. A figure is given of the germs contained in a network of fibrils; these latter are also shown in fibrin obtained by washing from the blood of a bird; and this fibrin is pervaded by particles like the nuclei of the blood-discs.—G. Gulliver, f.r.s.; *Phil. Mag. for Aug.* 1842.

---

Chemical Characters of the Spermatozoa, Molecules of the Semen. In a paper read at the Zoological Society, July 26, 1842, Mr. Gulliver stated that, in his experiments, the spermatozoa of mammalia were but little, or not at all, affected by nitric, muriatic, acetic, oxalic, tartaric, and citric acids; while the *spiral* spermatozoa of birds were very susceptible of the action of the vegetable acids, although the *cylindrical* spermatozoa of birds were nearly allied to those of mammalia in chemical characters; and that the chemical characters of the spermatozoa of man might probably be turned to account in medical jurisprudence.

The molecules of the semen he thinks may be connected with the perfecting of the semen, as they are to be found in small numbers at all times in that of healthy men, and in great abundance in that of birds and reptiles just before the testicles become ripe, and disappear or become scanty when the spermatozoa are completely formed. The molecules were described as resembling, in form and chemical characters, the minute particles which he has figured (Gerber's Anatomy,) in the juice of the supra-renal glands.

---

Millipedes discharged from the Human Stomach. A boy, fifteen years of age, had complained of stomachic derangement. At last he had an emetic of antimonial wine; after which he vomited a common-sized teacupful of woodlice, (millipedes,) an insect of the genus scolopendra. They were mostly alive and grown, but wanted the brown colour of those found in natural situations, being white. Their ova had been probably swallowed by the boy in his food. These insects often burrow in bacon.—Dr. A. W. Davis, Presteign; *Prov. Med. Journ.* No. xix. *Aug.* 13, 1842.

ON THE CHEMICAL ANALYSIS OF THE CONTENTS OF THE THORACIC DUCT IN THE HUMAN SUBJECT.—The author, availing himself of a favorable opportunity which presented itself of examining the contents of the thoracic duct in a human subject, procured an hour and a quarter after death by hanging, to the amount of six fluid drachms, obtained by analysis the following result:—

| | |
|---|---|
| Water, per cent. | 90·48 |
| Albumen, with traces of fibrinous matter | 7·08 |
| Aqueous extractive, or zomodine | 2·56 |
| Alcoholic extractive, or ozmazome | 0·52 |
| Alkaline chloride, carbonate and sulphate, with traces of phosphate, and oxide of iron | 0·44 |
| Fatty matters | 0·92 |
| | 100· |

The fatty matters possessed the same general characters as those of the blood, except that they did not contain phosphorus, as appeared from their yielding an alkaline, instead of an acid ash by incineration. The aqueous extractive differed from that of the blood by giving a ferruginous ash. The salts obtained by incineration from the alcoholic extractive yielded a larger proportion of alkaline carbonate than those of the blood. The author is confirmed, by the experiments he made on the present occasion, in his former views concerning the cause of the white colour of the chyle, which he ascribes to the presence of opaque white salivary matter as one of its constituents. The author then gives the results of his microscopical examination of the globules of the chyle, which he finds differ totally from those of the blood. He points out as being remarkable the large quantity of fatty matter existing in the chyle, and constituting an hydrocarbonaceous ingredient, which is constantly being added to the mass of blood, and is very rapidly consumed, as appears from the small quantity of this matter discoverable in the blood itself. The proportional quantity of ozmazome in the chyle he finds greatly to exceed that contained in the blood.—GEORGE OWEN REES, M.D.; *Proceedings of the Royal Society,* No. 52, *Feb.* 10, 1842.

---

EXTROPHY OF THE BLADDER. The hypogastrium was occupied by a tumour of a deep red colour, the size of a half section of a moderately large orange. Its surface was uneven and undulated, with several nodules; on each side of the inferior one were the orifices of the ureters. A thin, reddish fluid oozed from the whole surface; and, on irritating the orifices of the ureters with a probe, a small stream was ejected to a considerable distance. At the inferior part of the nymphæ was an opening, which resembled the urethra, but was, in reality, the vagina. There were neither clitoris or urethra. The child died seventeen days after birth, of cellular inflammation, which, commencing in the right leg, extended to every part of the body.

The pubis on each side was deficient. A prolapsis ani existed to some extent. During life the child had almost continual forcible straining. Was this owing to the deficiency of the pubis?

The peculiarities in this case were, its occurrence in a female, the undulated appearance of the vesical tumour, and the absence of the clitoris and urethra.—T. HARRISON, ESQ.; *Lancet, June* 25, 1842.

---

OF THE ULTIMATE DISTRIBUTION OF THE AIR-PASSAGES, AND OF THE MODES OF FORMATION OF THE AIR-CELLS OF THE LUNGS. After reciting the various opinions which have prevailed among anatomists regarding the manner in which the bronchial tubes terminate, whether, as some suppose, by cells having free communication with one another, or, as others maintain, by distinct and separate cells having no such intercommunication, the author states that having been engaged in investigating, with the aid of the microscope, the seat and nature of pulmonary tubercles, he could never discover, in the course of his enquiry, any tubes ending in a *cul-de-sac;* but, on the contrary, always saw, in every section that he made, air-cells communicating with each other. He concludes from his experiments and observations, that the bronchial tubes, after dividing dichotomously into a multitude of minute branches, which pursue their course in the cellular interstices of the lobules, terminate, in their interior, in branched air-passages, and in air-cells which freely communicate with one another, and have a closed termination at the boundary of the lobule. The apertures by which these air-cells open into one another are termed by the author *lobular passages:* but he states that the air-cells have not an indiscriminate or general intercommunication throughout the interior of a lobule, and that no anastomoses occur between the interlobular ramifications of the bronchiæ themselves; each branch pursuing its own independent course to its termination in a closed extremity. Several drawings of the microscopical appearances of injected portions of the lungs accompany this paper.—William Addison, f.l.s., Surgeon, Great Malvern; *Proceedings of the Royal Society,* No. 53, *March* 17, 1842.

---

Relative Sizes of the Trunks and Branches of Arteries. Ratio of the area of each arterial trunk to the joint area of its branches, or of its branches and continuation:

| | Trunk. | | Branches. |
|---|---|---|---|
| Arch of the aorta . . . | 1 | : | 1·055 |
| Innominata . . . | 1 | : | 1·147 |
| Common carotid . . . | 1 | : | 1·013 |
| External carotid . . . | 1 | : | 1·19 |
| Subclavian . . . | 1 | : | 1·055 |
| Abdominal aorta to the last lumbar arteries | 1 | : | 1·183 |
| Abdominal aorta just before dividing . | 1 | : | ·893 |
| Common iliac . . . | 1 | : | ·982 |
| External iliac . . . | 1 | : | 1·15 |

From this it follows that the notion of earlier anatomists, that the arterial canal enlarges as trunks divide into branches, is true, though the enlargement is less than was supposed. There is, however, one constant exception to the law now stated: namely, where the aorta divides into the common iliac arteries; for there, or at the division next lower down, the stream is always contracted.

The effect of such an arrangement must be to increase the velocity of the current, not only in the iliac arteries themselves, but in the arteries given off from the trunk above them, such as the mesenteric of the renal; and it is, surely, not improbable that the acceleration of the circulation through the kidneys, and through the organs from which the roots of the portal vein are derived, is the special purpose which so singular an arrangement serves.—James Paget, Esq., of St. Bartholomew's Hospital; *Medical Gazette, July* 8, 1842.

---

The Period of Puberty in Negro Women. The author maintains that the notion which generally prevails, of puberty being earlier in the negro than in the white races, and in hot than in temperate climates, is "no better than a vulgar error." He also regards as equally unfounded the opinion generally entertained, that certain mental and physical influences—as the modes of life of the richer and more luxurious classes, and working in heated factories—have an effect of hastening puberty; and affirms that period to be as early with peasants as with the inhabitants of cities.—John Robertson, Esq., Manchester; *Medical Gazette, July* 29, 1842.

---

On Corpora Lutea. Properly defined, the corpus luteum is a Graafian vesicle; and a Graafian vesicle is simply the receptacle for the nucleus of a cell which is contained within a nucleus or parent cell. Anatomically, the parts constituting the ovary and Graafian vesicle may be defined as one and indivisible; physiologically, the ovarium is the primitive body.

The Graafian vesicles possess two separate tunics, the outer one being vascular, the inner one not so. Dr. Lee first made known the fact that these bodies become more vascular during the menstrual period. It is difficult to state with precision the period at which solid matter begins to form in the Graafian vesicle. The cavity may be filled with blood, a serous, straw-coloured fluid, or simply the granular reliquiæ of the ovum. The solid matter of the corpus luteum, examined microscopically, appears to consist neither of secretion or effusion; but to be a true growth, made up of two separate parts. The first, small, nucleated cells, common to all parts of the body; the second consists of cells also, but much enlarged, floating freely, and filled with small granules.

The purpose served by the corpus luteum it is not easy to ascertain; but, perhaps, the best explanation which can at present be given is, that the escape of "a substance" having caused a breach in the ovary, the corpus luteum, by filling up the aperture, brings the sides of the cavity into apposition, and thus heals the lesion. This purpose being accomplished, the office of the corpus luteum is fulfilled, and the absorbents remove it.

The locality of the corpus luteum is very palpable. It is invariably interposed between the external or vascular membrane of the Graafian vesicle, and the internal or non-vascular ovisac, which is always more or less thickened, and has a greater or less quantity of granular or other matter internal to it, in pure specimens. The structure is decidedly lobular, having a radiated appearance; one of the radii always extending as far as that portion of the ovary, whence the ovum originally made its escape.—FRANK RENAUD, Esq., Edinburgh; *Lon. and Edin. Monthly Journ. of Med. Science*, No. vi. *June*, 1842.

---

INFLUENCE OF THE CORONARY CIRCULATION ON THE ACTION OF THE HEART. By a variety of experiments on the hearts of dogs and rabbits, in which the animals were pithed, the thorax opened as quickly as possible, and ligatures applied to the coronary arteries, Mr. Erichsen found that the action of the heart ceased considerably sooner than when no ligatures were applied. In an animal that is pithed, but the heart of which is uninterfered with, cardiac pulsation will go on for an hour and half, under the influence of artificial respiration, but only, at an average, for about twenty-three minutes and half after ligature of the coronary arteries, and about thirty-two minutes and forty seconds after the death of the animal. He therefore agrees with Dr. Hall in thinking that ossification of the coronary arteries, a fatty condition of the heart, a contracted aortal, or deficient nitral valve may, by interrupting the flow of blood through the coronary vessels, give rise to syncope or sudden dissolution.—JOHN E. ERICHSEN, Esq. *Medical Gazette, July* 8, 1842.

---

ON THE FORCES BY WHICH THE BLOOD IS CIRCULATED IN THE CAPILLARY VESSELS. The author, in a somewhat elaborate paper, endeavours to show that the capillaries have an important influence in carrying on the circulation of the blood.

He strongly objects to the experiments of Dr. Hall and other physiologists, in relation to the functions of the capillaries, that in most or all of these experiments the mutual relations uniting in harmonious action the several powers carrying on the circulation, are destroyed. When, for example, a ligature is passed round the limb of an animal, or when the aorta is tied, or the heart itself destroyed, in each of these cases the circuit of the circulation is broken; and it necessarily and obviously follows that the parts below the ligature (in the case of ligature) cannot receive blood, on the one hand, nor the parts above the ligature propel it, on the other.

Sensible that all such experiments are fraught with insuperable difficulties, the author set himself to devise some method by which the action of the capillaries might be determined with precision, and the placenta appeared to him to be the most proper organ for the suitable observations and experiments. The whole substance of it may be regarded as composed of arteries, veins, and capillaries; and although the disengagement of it from the uterus disturbs the normal relation of those vessels, it appeared to the author, notwithstanding, possible to obtain sufficiently accurate results. Accordingly, a placenta was procured twenty minutes after separation from the uterus, and placed, with the exception of the cord, in a bladder, which was immersed in water, at the temperature of 100° Fahrenheit. The free extremity of the cord at the same moment was elevated to an angle of 30°, resting on the edge of a glass, and at the distance of a foot from the placenta. At the commencement of the experiment no blood escaped from the vein, but in two minutes from the immersion of the placenta the blood began to flow, and continued to do so for about twenty minutes; and at this time it was found that the glass had received about an ounce. The author regards this experiment as less exceptionable and more conclusive than those usually referred to, in regard to the circulatory function of the capillaries.—Dr. Calvert Holland, Sheffield; *Edin. Med. and Surg. Journ., July* 1, 1842.

